# Idiopathic sclerosing dacryoadenitis

**DOI:** 10.1186/s12348-023-00365-y

**Published:** 2023-09-19

**Authors:** Samantha D. Butterfield, Rona Z. Silkiss

**Affiliations:** 1 Department of Ophthalmology, California Pacific Medical Center, Suite 250, San Francisco, Van Ness, CA 94102 USA; 2 Silkiss Eye Surgery, 400 29Th Street, Suite 315, Oakland, CA 94609 USA

## Abstract

Dacryoadenitis is an inflammation of the lacrimal gland. This condition has an extensive differential diagnosis, requiring a thorough workup to identify the underlying etiology. If no etiology is identified, the condition is termed idiopathic dacryoadenitis. The purpose of this report is to present a case of idiopathic sclerosing dacryoadenitis and review the diagnostic process.

We present a case of sclerosing dacryoadenitis non responsive to systemic antibiotics and steroids, improving after surgical debulking/biopsy. Systemic inflammatory and infectious labs were negative. Tissue was negative for SARS-CoV-2 antigen. Histopathologic review of the surgical specimen revealed nonspecific, sclerosing dacryoadenitis, ultimately supporting the diagnosis of idiopathic nonspecific fibrosing dacryoadenitis.

This case reviews the presentation, evaluation, and management of a common orbital pathologic condition, with updated recommendations based on the most current literature.

## Background

Dacryoadenitis is an inflammation of the lacrimal gland with an extensive differential diagnosis, including inflammatory, infectious, and malignant disease. Common presenting signs include edema, erythema, and tenderness overlying the superolateral orbit. Further examination may reveal conjunctival injection, ptosis, proptosis, and ocular motility defects. Systemic symptoms such as weight loss, fatigue, lymphadenopathy, or fever may be present, suggesting a more sinister underlying systemic etiology [1, 2]. The differential for dacryoadenitis is broad, including infectious, inflammatory, and neoplastic. In many cases of inflammatory dacryoadenitis, the underlying etiology remains unknown and is thus labeled idiopathic. Idiopathic lacrimal gland inflammation can be classified into lymphoid follicular, sclerosing, mixed, or lymphocytic infiltration, with each class representing a distinct inflammatory process [3, 4]. Understanding of the etiology of this rare disease is evolving and reliant on clinical evidence and practical considerations. In this paper, we present an interesting case of idiopathic sclerosing dacryoadenitis with implications for practical diagnosis of this poorly understood disease.

## Main text

### Case

**Fig. 1:**
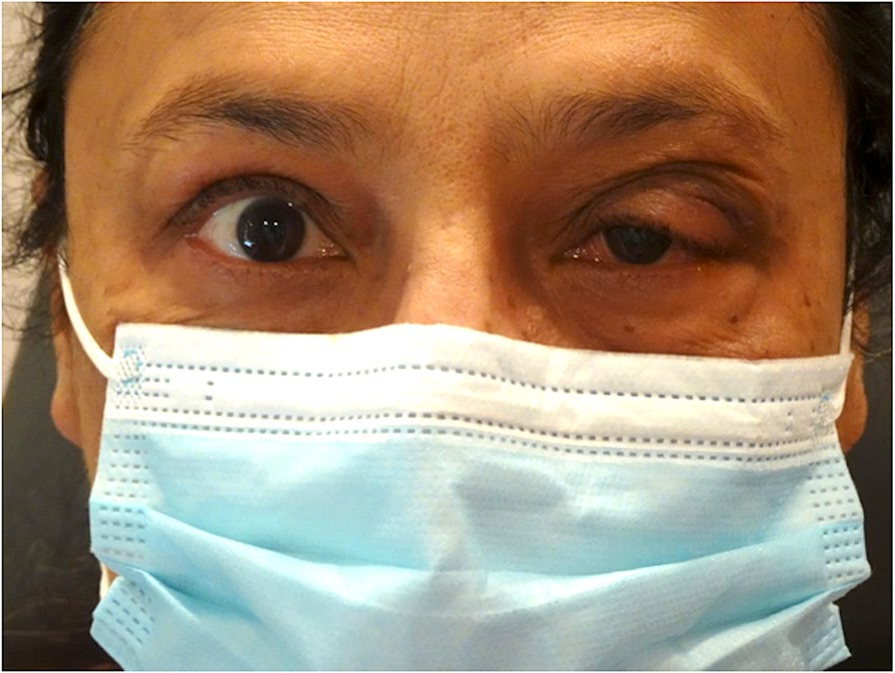
Initial presentation with left upper eyelid edema and erythema

A female in her 60 s with a past medical history of type 2 diabetes, hyperlipidemia, and hypertension presented to our clinic at the referral of her ophthalmologist with several months of progressive swelling, pain, and redness of her supertemporal eyelid despite completing a 10 day course of Augmentin (Fig. 1). Ophthalmic examination was notable for a tender, firm, palpable mass in the area of the left supertemporal orbit with associated brawny edema. Visual acuity, intraocular pressure, color vision, pupils, and a comprehensive examination were otherwise normal. The patient was given a 14-day course of doxycycline and a methylprednisolone dose pack, and laboratory studies were obtained.

**Fig. 2:**
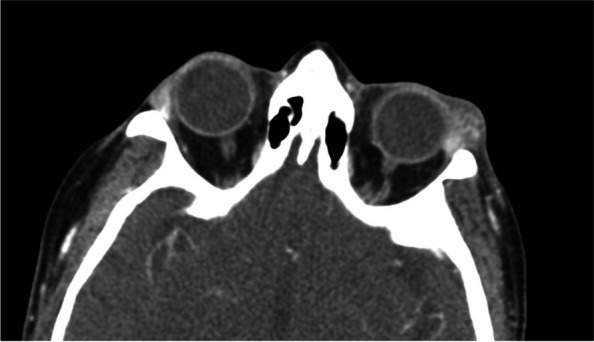
Axial CT scan of the orbits depicting left sided lacrimal gland enlargement

Evaluation of complete blood count, erythrocyte sedimentation rate, C-reactive protein, antinuclear antibodies, angiotensin converting enzyme, anti-Sjogren’s syndrome A, anti-Sjogren’s syndrome B, rheumatoid factor, and immunoglobulin G were all normal. Perinuclear anti-neutrophil cytoplasmic antibodies were elevated, but myeloperoxidase anti-neutrophil cytoplasmic antibodies and proteinase-3 antibodies were normal. CT revealed left lacrimal gland enlargement (Fig. 2).

**Fig. 3:**
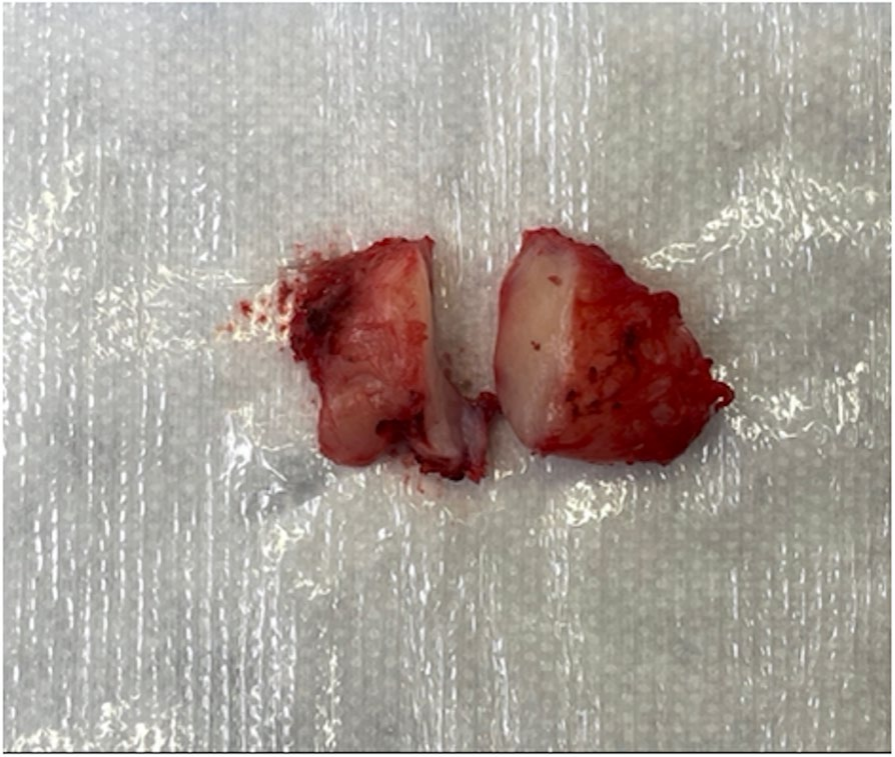
1.7 × 0.8 × 0.7 cm, grossly rubbery and fibrosed lacrimal gland specimen

Symptoms did not improve with the oral antibiotics and steroids. Lacrimal gland biopsy was recommended. The patient underwent a left anterior orbitotomy with biopsy and removal of the mass, capsule intact. A 1.7 × 0.8 × 0.7 cm, grossly rubbery and fibrosed specimen (Fig. 3) was sent for fresh and formalin histopathological evaluation as well as flow cytometry and immunohistochemistry.

**Fig. 4:**
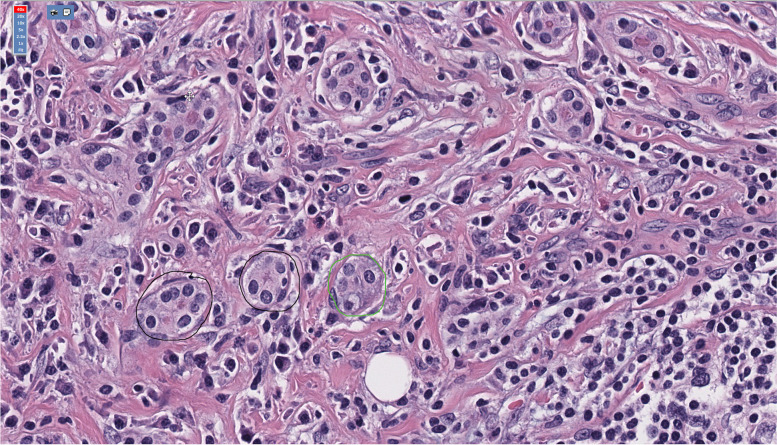
Histologic slide of tissue from the surgical specimen stained with hematoxylin and eosin viewed at 40 × magnification. Notice residual acini (outlined) separated by fibrosis and inflammation comprised predominantly of plasma cells and lymphocytes. The edge of a lymphoid follicle is visible in the lower right corner

**Fig. 5:**
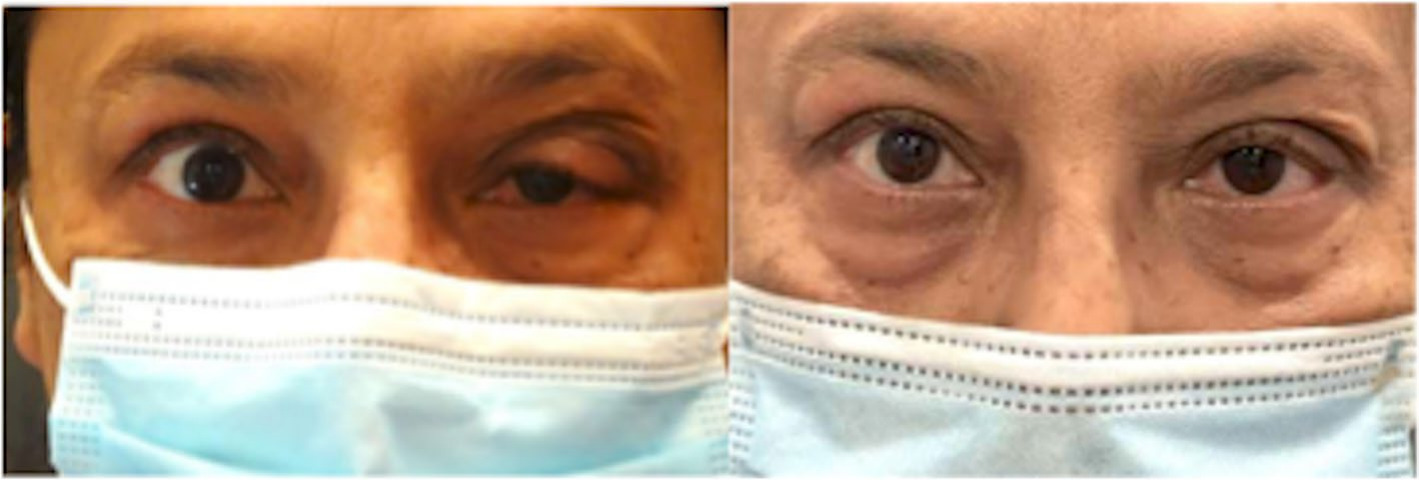
External photos before (left) and after (right) surgical biopsy/debulking

Tissue analysis revealed an extensive, diffuse fibro-inflammatory process extending between acini and into adjacent stroma, with fibroblasts forming fascicles without a well-defined pattern (Fig. 4). The inflammatory component consisted of plasma cells, lymphocytes, and rare eosinophils. Immunohistochemistry revealed 15% immunoglobulin G4 to immunoglobulin G ratio, not consistent with Immunoglobulin G4 Related Disease. Flow cytometry revealed a predominance of lymphocytes, with no immunophenotypic evidence of a lymphoproliferative disorder or plasma cell neoplasm. Tissue was then sent for additional testing to evaluate for COVID-19 related inflammatory disease. SARS-CoV-2 T62 immunostaining, in situ hybridization of SARS-CoV-2 CovSpike gene expression, and sequencing for SARS-CoV-2 RNA were negative. Histopathologic studies ultimately supported a diagnosis of nonspecific fibrosing dacryoadenitis. The patient’s signs of inflammation and pain disappeared within several weeks after surgery (Fig. 5).

## Discussion

Dacryoadenitis in an inflammation of the lacrimal gland that presents with pain, redness, and swelling of the superolateral eyelid. It is typically unilateral and occurs mostly in children and young adults. On exam there may be hyperemia of the palpebral lobe of the lacrimal gland, chemosis, ipsilateral preauricular lymphadenopathy, and/or fever. The differential diagnosis of this presentation includes hordeolum, preseptal cellulitis, orbital cellulitis, idiopathic orbital inflammation, primary or metastatic malignancies, retained foreign body, and, in children, dermoid cyst or rhabdomyosarcoma [5].

Inflammatory non-infectious, viral, and bacterial etiologies of dacryoadenitis exist. Viral etiologies include mumps, mononucleosis, influenza, and varicella zoster. Bacterial dacryoadenitis is rare but has been reported and is usually due to staphylococcus aureus, Neisseria gonorrhoeae, or streptococci [5]. Mycobacterial disease should also be considered. The most common etiology of dacryoadenitis is inflammatory, non-infectious disease [1]. Onset may be acute or indolent. In the majority of patients, a systemic etiology is identified. Common culprits include Sjogren’s Syndrome, Sarcoidosis, Granulomatosis with Polyangiitis, and Immunoglobulin G4 Related Disease (IgG4RD) [1].

All patients with a presentation concerning for dacryoadenitis should undergo a thorough history and ophthalmic exam including Hertel exophthalmometry, extraocular motility assessment, parotid gland and cervical lymph node palpation, and vitals including temperature. Any ocular discharge should be smeared and cultured. CT or MRI of the orbits should be performed preferably with contrast. Laboratory evaluation should include a CBC with differential, erythrocyte sedimentation rate, c-reactive protein, antinuclear antibodies, angiotensin converting enzyme, anti-Sjogren’s syndrome A and B antibodies, rheumatoid factor, and immunoglobulin G antibodies with immunoglobulin G-4 ratio.

Since the outbreak of the coronavirus (SARS-CoV-2) in 2020, the ophthalmic community continues to assess the implications of infection with this virus on new and existing disease processes. Recent literature suggests that the physician consider SARS-CoV-2 when evaluating the patient with dacryoadenitis. Recent research out of Hong Kong suggests that SARS-CoV-2 infection is associated with an increased risk of developing various autoimmune disease, and that the risk could be attenuated by COVID-19 vaccination [6]. In their retrospective case–control study, Kase et al. compared lacrimal gland tissue between two middle-aged women with idiopathic dacryoadenitis, one of whom was also positive for SARS-CoV-2 [7]. They found that in the woman infected with the coronavirus there was immunoreactivity for SARS-CoV-2 nucleocapsid protein and strong angiotensin converting enzyme-2 (ACE2) expression in the lacrimal gland. In the woman without coronavirus infection SARS-CoV-2 nucleocapsid protein immunoreactivity was not observed, but ACE2 was expressed in the gland. Martinez Diaz et al. described a case of idiopathic dacryoadenitis associated with recent SARS-CoV-2 infection [8]. Senol Kobak, in a recent letter to the editor of Rheumatologia, reported a case of reactivation of IgG4RD secondary to SARS-CoV-2 infection [9]. In addition to COVID infection, COVID vaccination has also been implicated in the development of dacryoadenitis. Murphy et al. recently published (BMJ Case Reports) the case of a 14-year-old boy who nine hours after his first COVID vaccination (Pfizer-BioNTech) developed acute onset unilateral dacryoadenitis with normal laboratory studies and CT confirming enlargement of the lacrimal gland [10]. Whether SARS-CoV-2 active infection, previous infection, immunization, or all of these contribute to the development of dacryoadenitis remains to be proven. However, the growing body of observations warrant attention to this virus when evaluating a patient with dacryoadenitis. In addition to thorough history taking and widely available SARS-CoV-2 antigen and antibody testing, lacrimal gland tissue from biopsy can be sent for further workup (discussed in more detail in the next paragraph).

Biopsy can be a valuable diagnostic and therapeutic tool in managing dacryoadenitis. Cases with suspected systemic involvement, or those resistant to treatment with steroids and antibiotics, should be considered for biopsy. Biopsy with histopathologic review is often the only way to identify the etiology of dacryoadenitis. When performing a lacrimal gland biopsy, one aims to leave the palpebral lobe of the gland intact, protecting the lacrimal ductules. However, ultimately the biopsy must be performed in the area of pathology regardless of lobe involvement. Debulking or complete excision can be done, depending on the quality of the tissue and the expected diagnosis. When malignancy is expected, extra care must be taken when performing a biopsy (the details of which are outside the scope of this report). Once obtained, the tissue should be sent for fresh and frozen pathologic evaluation and flow cytometry. Evaluation for coronavirus-related disease should be done with tissue SARS-CoV-2 T62 immunostaining, in situ hybridization of SARS-CoV-2 CovSpike gene expression, and sequencing for SARS-CoV-2 RNA.

In roughly one third of cases, an etiology for dacryoadenitis is never identified and is thus diagnosed as idiopathic dacryoadenitis [1]. Idiopathic dacryoadenitis is the presenting sign of Idiopathic Orbital Inflammation (IOI) in up to 40% of cases [1]. Histopathological review of biopsies can aid in diagnosis, but still many cases remain idiopathic. The etiology of idiopathic dacryoadenitis or even IOI may be clarified as PCR testing for a variety of pathogens is increasingly undertaken in the course of diagnosis.

In a study of 79 cases of idiopathic dacryoadenitis, Andrew et al. found 56% of biopsies demonstrated classic lymphoid infiltrate, while 29% demonstrated sclerosing infiltrate, such as that seen in the patient reported above [11]. Little is known about sclerosing idiopathic dacryoadenitis, but Andrew et al. observed that sclerosing cases had more insidious onset, longer symptom duration after biopsy, and more incomplete treatment response. Previous studies have reported lacrimal gland fibrosis at various times in the disease course, supporting the theory of sclerosing dacryoadenitis as a primary disease process rather than a histopathologic finding of later stages of the disease [3, 4].

Management of dacryoadenitis depends on the suspected etiology. When the etiology is unclear, it is recommended to treat empirically with systemic antibiotics for 24–48 h followed by reassessment [5]. Viral etiology can often be managed with supportive care. Inflammatory, non-infectious dacryoadenitis is typically treated with oral corticosteroids [1]. Treatment with radiation therapy or systemic immunomodulating therapy has also been described [12, 13]. If a biopsy is to be done, then one should consider debulking at the time of surgery to aid in recovery.

Dacryoadenitis is a common orbital disease with a broad differential diagnosis that is further expanding in the post-coronavirus era. We report an interesting case of idiopathic sclerosing dacryoadenitis with diagnosis made by histopathology. Additionally, we provide a comprehensive yet concise review of the diagnostic and therapeutic process of caring for a patient with dacryoadenitis.

## Data Availability

Data sharing is not applicable to this article as no datasets were generated or analyzed during the current study.

## Abbreviation

SARS-CoV-2: Severe acute respiratory syndrome coronavirus 2
